# Oral rehydration therapy and Zinc treatment among diarrhoeal children in India: Exploration from latest cross-sectional National Family Health Survey

**DOI:** 10.1371/journal.pone.0307657

**Published:** 2024-10-03

**Authors:** Shiva S. Halli, Rajeshwari A. Biradar, Jang B. Prasad

**Affiliations:** 1 Department of Community Health Sciences, Institute for Global Public Health, University of Manitoba, Winnipeg, Manitoba, Canada; 2 Shri Murughendr Shivayogi Vishvast Vidyapeeth M G C G Memorial College, Athani, Belgaum, India; 3 Jawaharlal Institute of Postgraduate Medical Education & Research, Puducherry, India; National Research Centre, EGYPT

## Abstract

**Background and aims:**

Diarrhoea is one of the deadliest diseases and causing death among children in India, but no systematic attempt is made to understand it especially its control using oral rehydration salts (ORS). It is well known that use of ORS and Zinc have been effective in containing diarrhoea among children. An attempt is made using large scale national data set in India to understand use of ORS and Zinc to control diarrhoea and their associated factors among diarrhoeal children under five in India.

**Methods:**

Publicly available most recent cross-sectional National Family Health Survey data in India was used for the study. The multi-stage cluster sampling design was used with 2011 Census of India as a sampling frame. Households were selected using a Systematic Random Sampling design from selected primary sampling units in rural and urban clusters. From the selected households, the eligible children were those who suffered from diarrhoea in the two weeks preceding the survey and were less than 5 years old. Using this criterion, out of 232,920 children in the survey of less than five years, 16,213 sample diarrheal children found to be available for the study. Both descriptive and inferential statistical techniques were used to analyse the data.

**Results:**

Across India, 61% and 31% of the children were given ORS and Zinc respectively. However, combined ORS + Zinc treatment was only around 24%. The treatment of ORS, Zinc, and combined ORS + Zinc supplementations were significantly higher among younger children, children of 24–35 age group mothers, children from rich wealth index groups, belong to Hindu religion and general caste compared to their counterparts. The logistic regression results showed that consumption of ORS among diarrhoeal children under five years of age depends upon size of a child at birth. For instance, diarrheal children who were very small size at birth compared to very large at birth, had 39% lower odds of consuming ORS (AOR = 0.61; CI 0.48, 0.78; p<0.001). Another important variable is place of first treatment sought for diarrheal children. That is children who first sought treatment in private hospital compared to government hospital had 52% lower odds of ORS consumption. The logistic regression adjusted AORs are similar with Zinc and ORS + Zinc supplementations.

**Conclusions:**

To improve the coverage and management of childhood diarrhoea in India, planning activities should focus not only on distribution, and increasing knowledge of ORS preparation especially for urban slum residents and rural disadvantaged groups through demonstration. There should be also proper focus on providing ongoing pathways to ensure proper supply chains.

## Introduction

Globally diarrhoeal disease is found to be the second leading cause of death among children in developing countries [1–3]. The review of literature has shown that nearly half a million children die every year from diarrhoea in Africa and Asia especially in South Asia [1, 4–6]. This has consequences in other spheres of life including negative impact on social and economic conditions. It makes sense for African and South Asian countries to prevent deaths among children using well known most effective treatment of oral rehydration solutions (ORS) and improve their socio-economic conditions [7, 8]. The improvement in socio-economic conditions is useful in scaling up effective interventions to prevent diarrhoeal deaths among children with the use of ORS and Zinc and improve health and well-being among children by providing with nutritious diet and improved hygiene [9–13]. International organizations such as World Health Organization (WHO) and the United Nations International Children’s Emergency Fund have been advocating use of ORS in preventing diarrhoeal deaths among children and Zinc in reducing severity and recurrence of diarrhoea at least for next 2 to 3 months [9–13].

India is making significant improvement in preventing diarrhoeal deaths among children especially in the last decade, however, India is still experiencing more diarrhoeal deaths among children compared to other countries in the World [14–16]. The Government of India along with the Indian Academy of Paediatrics have issued guidelines to treat diarrhoea with ORS and Zinc among children way back in 2006 [17]. To be more specific the guidelines include treatment of diarrhoea using ORS and 20mg Zinc supplementation among the new-borns for 14 days. In case of children from 2 to 6 months old, it was recommended that Zinc to be taken daily [17].

Despite India being the World leader in diarrhoea deaths among children, there are hardly any studies using large scale nationally representative data to understand the use of ORS and its correlates in preventing diarrhoea deaths among children. There are a few studies but tend to be either hospital based, small community based or limited regionally based [14, 18–21]. The most recent National Family Health Survey-V (2020–21) contains a national sample of 16,213 children under five suffering from diarrhoea two weeks prior to the survey and its treatment with ORS and zinc supplementations [22]. This is a great opportunity for researchers to address the prevalence, the treatment and correlates using the data set. Hence, the purpose of this study was to present the prevalence of use of ORS and Zinc supplementation in the treatment of diarrhoea disease among children under five in India. Using both descriptive and inferential statistics, an attempt is made to identity associated factors and hoping that this would help the Indian policy makers to focus on suitable facilitators and barriers to develop scalable effective interventions.

## Methods

The National Family Health Survey (NFHS) is a periodic survey conducted by the International Institute for Population Sciences, Mumbai, India using the multi-stage cluster sampling design. The data used for the analysis is from the fifth round of the NFHS, conducted in 2019–21. NFHS is a nationally representative cross-sectional survey including representative sample households throughout India. The survey provides state, national and district-level estimates of demographic, health, socioeconomic status, and program dimensions, which are critical for implementing the desired changes in demographic and health parameters. Stratified, two-stage sampling is primarily used in all NFHS surveys to obtain a representative sample of households. Probability proportional to size (PPS) was used to select the households from all states and Union Territories. Within each rural stratum, villages were selected from the sampling frame based on the 2011 Census with PPS. In urban areas, the Census Enumeration block (CEB) was selected based on data from the census of India. In the second phase, households were selected using a Systematic Random Sampling design from selected primary sampling units in rural and urban clusters. From the selected households, the respondents were selected. The survey represents all the districts of all the States and Union Territories of India. The household sample size in the survey consists of 636,699 households, and carefully selected well trained interviewers collected information from the respondents using a computer-assisted personal interviewing (CAPI) electronic device. All precautions were taken to make sure that the interview was conducted in a private place and confidentiality was maintained. For this study, the Kids file was used. Detailed information on the survey methodology, the design and the sampling plan are provided on the NFHS website [22].

### Ethical approval

The study used publicly available data set. Since it is a secondary analysis and hence, no ethical approval is required.

### Analytical sample

The eligible children were those who suffered from diarrhoea in the two weeks preceding the survey and were less than 5 years old. Using this inclusion criterion, out of 232,920 children in the survey of less than five years, 16,213 sample diarrheal children were found to be available for the study.

### Outcome variable

In this study we are interested in assessing the use of ORS supplementations in the treatment of diarrhoea among children those who suffered from diarrhoea in the two weeks (current episode) preceding the survey and were less than 5 years old. Hence, we have categorized the outcome variable as 1 if eligible children suffered from diarrhoea in the two weeks preceding the survey and 0 if children did not suffer from suffer from diarrhoea in the two weeks preceding the survey and were less than 5 years old.

### Predictor variables

The main predictor variable in this study was diarrhoea treatment supplementations such as ORS, Zinc and combined ORS + Zinc. In assessing the significance of background factors in the use of ORS supplementations in the treatment of diarrhoea among children were type of health facility, mothers age, mother’s educational status, type of residence, regions of India, caste, religion, and wealth Index. The wealth Index is a composite measure, and it was constructed by the survey authorities and was provided in the publicly available data set [22]. Other predictors were currently breastfeeding or not, sex of child, birth weight of a child at birth, age of child (in months) and diarrhoea in the last two weeks and food supplements.

### Data analysis

Data analysis consisted of assessing both bivariate and multivariate associations between diarrhoea treatment supplementations such as ORS, Zinc and combined ORS + Zinc, and the background characteristics. The bivariate analysis was useful not only in assessing the significance of background factors in the use of ORS supplementations in the treatment of diarrhoea among children and in selecting background characteristics for a binary logistic regression analysis. While conducting logistic regression analysis, in case of a categorical explanatory variable, dummy variables were created with the appropriate reference category. The results of logistic regression for explanatory variables included adjusted odds ratios, 95% confidence intervals and level of significance. The weighted analysis was conducted using SPSS software version 20.

## Results

### Background characteristics

**Fig 1:**
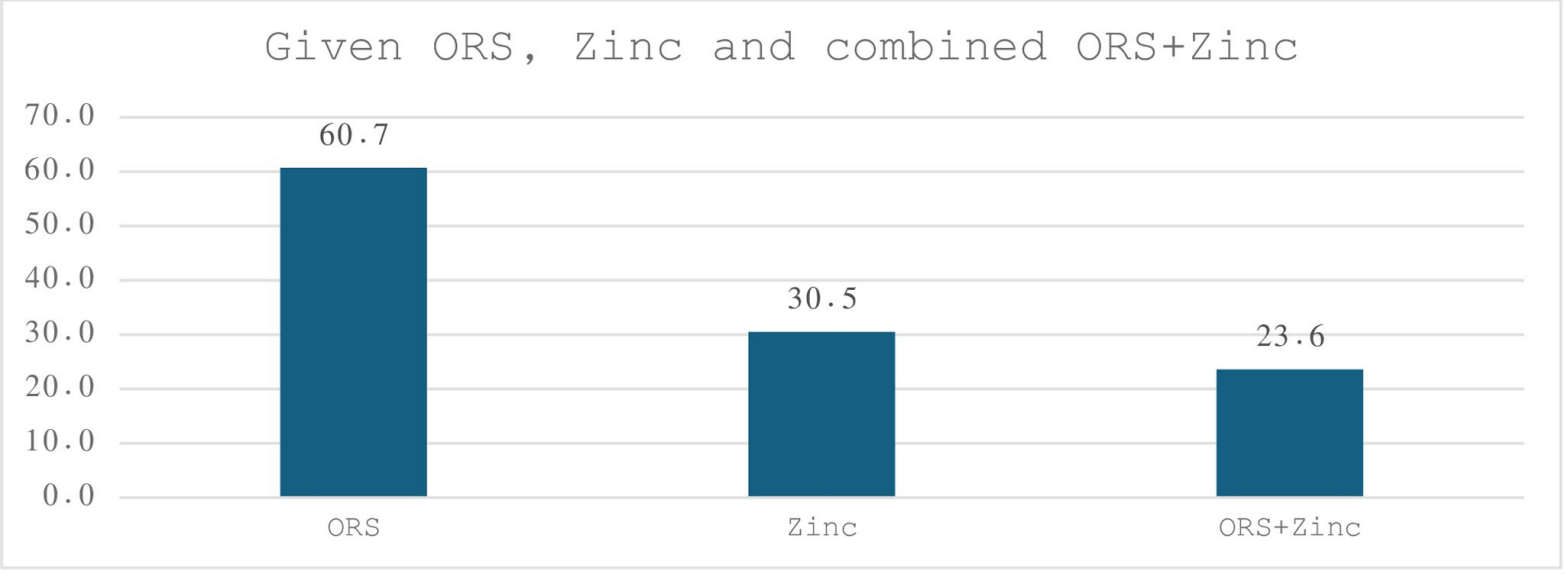
Prevalence of ORS, Zinc and combined ORS + Zinc supplementations among diarrhoeal children in India, NFHS-V.

The number of children under five years who suffered from diarrhoea in the past two weeks were 16,213 according to the survey. The sociodemographic characteristics of the children and their mothers were presented in Table 1. More than 31% children were less than 12 months old, and 23.9% of the children were between 13–23 months. Around two third of children at birth were average size with 2.8 kilogram. Most children received first treatment for diarrhoea in private facilities, and about 31.5% and 36% children were offered somewhat less amount to eat and drink during the diarrhoea weeks. Diarrhoeal Children from urban and rural areas were 22.7% and 77.3%, respectively. Treatment with ORS, Zinc and combined ORS + Zinc supplementations were provided to 60.7%, 30.5% and 23.6% of the diarrhoeal children respectively (Fig 1).

**Table 1:** Background characteristics of diarrhoeal children in India, 2019–21.

Background characteristics	Categories	%	N
Age of Mother	15–24	40.7	6593
	25–34	52.5	8510
	35+	6.8	1111
Highest educational level	No education	22.4	3632
	Primary	13.3	2150
	Secondary	52.7	8537
	Higher	11.7	1894
Type of place of residence	Urban	22.7	3682
	Rural	77.3	12531
Wealth index	Poorest	29.6	4791
	Poorer	23.9	3868
	Middle	19.4	3146
	Richer	16.3	2648
	Richest	10.9	1759
Currently breastfeeding	No	27.3	4426
	Yes	72.7	11787
Sex of child	Male	53.6	8698
	Female	46.4	7515
Birth weight	Very large	8.5	1369
	Large	12.5	2010
	Average	66.0	10616
	Low birth weight	9.4	1516
	Very low birth weight		
		3.6	574
Age of child (in months)	> = 12	31.2	5023
	13–23	23.9	3846
	24–35	18.2	2923
	36–47	14.4	2314
	48–59	12.3	1975
Caste	Scheduled caste	25.2	3889
	Scheduled tribe	11.0	1689
	Other Backward Castes	44.6	6873
	None of above	19.2	2952
Religion	Hindu	79.6	12910
	Muslim	16.7	2705
	Others	3.7	598
Birth order	1	37.4	6066
	2	33.1	5368
	3	16.1	2613
	4+	13.4	2167
Indian regions	North	11.4	1797
	Central	21.6	3422
	East	38.6	6107
	Northeast	3.0	483
	West	15.2	2402
	South	10.2	1615
Place first sought treatment for diarrhoea	Government	24.4	3950
	Private	50.1	8115
	Others	25.6	4148
Diarrhoea in last 2 weeks: amount offered to drink	Nothing to drink	4.6	742
	Much less	23.1	3717
	Somewhat less	36.1	5811
	About the same	30.8	4968
	More	5.4	868
Diarrhoea in last 2 weeks: amount offered to eat	Stopped food	1.7	272
	Never gave food	7.6	1216
	Much less	24.1	3882
	Somewhat less	36.2	5833
	About the same	27.7	4452
	More	2.8	445

### Associated factors for diarrhoeal disease

Bivariate associations between background characteristics and diarrhoeal children treatment with ORS, Zinc and ORS + Zinc combined presented in Table 2. Most of the variables did not show significant associations. The only variables those showed some variations in the treatment of diarrhoea with ORS, Zinc and ORS + Zinc combined were age of the mother, age of children, size of child at birth and amount of food and drink those were provided during the disease period. These are all positive associations. To be more specific, mothers with age groups 15–24, 25–34 and 35+ and the corresponding percentage of children with ORS treatment for diarrheal children were 57%, 63% and 67% respectively. Similarly, categories of size of children such as very large, large, average, smaller than average and very small and the corresponding percentage of children with ORS treatment for diarrheal children were 72%, 64%, 59%, 61% and 60% respectively. Similar associations between background characteristics and diarrheal children treatment with Zinc and ORS + Zinc were found though the percentages of treated children were less than half of percentages of diarrheal children treated with ORS.

**Table 2:** ORS, Zinc and combined ORS + Zinc consumption by background characteristics of diarrhoeal children in India, 2019–21.

		ORS %	Total	Zinc %	Total	ORS + Zinc %	Total
Age of Mother	15–24	56.6	6586	29.1	6593	21.0	6586
	25–34	63.1	8494	31.6	8510	25.3	8494
	35+	67.1	1109	31.2	1111	25.5	1109
Highest educational level	No education	59.8	3624	27.4	3632	21.4	3624
	Primary	58.2	2147	29.4	2150	22.4	2147
	Secondary	61.7	8527	31.7	8537	24.2	8527
	Higher	61.0	1890	32.8	1894	25.9	1890
Type of place of residence	Urban	62.5	3681	31.5	3682	24.8	3681
	Rural	60.2	12508	30.3	12531	23.2	12508
Wealth index	Poorest	59.4	4783	28.3	4791	21.7	4783
	Poorer	59.5	3861	29.8	3868	22.4	3861
	Middle	60.7	3142	31.5	3146	24.0	3142
	Richer	62.6	2645	31.6	2648	24.5	2645
	Richest	64.2	1758	35.1	1759	29.2	1758
Currently breastfeeding	No	63.2	4418	32.3	4426	25.4	4418
	Yes	59.8	11770	29.9	11787	22.9	11770
Sex of child	Male	61.9	8687	30.9	8698	24.1	8687
	Female	59.4	7502	30.2	7515	22.9	7502
Birth weight	Very large	72.1	1367	45.1	1369	38.0	1367
	Large	64.3	2006	30.8	2010	24.1	2006
	Average	58.8	10604	28.9	10616	22.0	10604
	Low birth weight Very low birth weight	61.4	1514	29.2	1516	22.4	1514
		59.9	571	30.3	574	22.5	571
Age of child (in months)	> = 12	49.8	5017	26.8	5023	18.4	5017
	13–23	66.7	3840	31.2	3846	25.5	3840
	24–35	64.2	2919	31.5	2923	24.9	2919
	36–47	66.1	2308	33.3	2314	26.2	2308
	48–59	65.1	1973	34.0	1975	27.7	1973
Caste	Scheduled caste	58.6	3885	31.2	3889	24.2	3885
	Scheduled tribe	65.8	1687	33.3	1689	26.1	1687
	Other Backward Castes	58.1	6866	29.9	6873	22.4	6866
	None of above	65.1	2942	28.2	2952	22.5	2942
Religion	Hindu	60.5	12891	30.8	12910	23.8	12891
	Muslim	62.5	2701	29.5	2705	23.1	2701
	Others	56.6	597	28.7	598	21.2	597
Birth order	1	59.4	6056	30.7	6066	23.2	6056
	2	61.4	5362	32.3	5368	25.2	5362
	3	61.2	2606	29.3	2613	23.0	2606
	4+	62.1	2164	26.9	2167	21.4	2164
Indian regions	North	62.6	1795	29.1	1797	22.9	1795
	Central	55.2	3420	30.8	3422	23.0	3420
	East	61.7	6092	28.7	6107	22.4	6092
	Northeast	69.7	481	29.2	483	24.1	481
	West	61.8	2401	30.0	2402	23.5	2401
	South	63.1	1614	37.5	1615	29.5	1614
Place first sought treatment for diarrhoea	Government	76.3	3946	41.1	3950	34.4	3946
	Private	61.7	8103	31.3	8115	23.8	8103
	Others	43.9	4140	19.0	4148	12.9	4140
Diarrhoea in last 2 weeks: amount offered to drink	Nothing to drink	46.4	740	23.3	742	15.0	740
	Much less	65.5	3717	32.2	3717	25.1	3717
	Somewhat less	61.1	5802	31.8	5811	24.5	5802
	About the same	57.6	4959	29.4	4968	22.5	4959
	More	69.8	868	29.1	868	25.0	868
Diarrhoea in last 2 weeks: amount offered to eat	Stopped food	51.9	272	27.0	272	21.6	272
	Never gave food	41.7	1213	22.1	1216	12.9	1213
	Much less	66.6	3880	31.7	3882	25.1	3880
	Somewhat less	62.7	5825	32.0	5833	25.0	5825
	About the same	58.8	4445	30.3	4452	23.5	4445
	More	64.6	445	31.3	445	25.3	445

### Logistic regression analysis

The logistic regression analyses were conducted separately for three types of treatment for diarrheal children such as ORS, Zinc and ORS + Zinc combined. Results presented in Table 3 showed that consumption of ORS, Zinc, and combined ORS + Zinc supplementations for diarrhoeal disease treatment of children were significantly associated with mothers’ age, age of children, place of first treatment sought for diarrhoea, size of a child at birth and amount of food offered to eat and drink during diarrhoea after adjusting for the confounding characteristics, and direction of associations were similar to what we observed in Table 2. To be more specific, the logistic regression results showed that consumption of ORS among diarrhoeal children under five years of age, 20% higher adjusted odds ratio among children of mothers who were 25 to 34 years compared to children of mothers who were 15 to 24 years of age (AOR = 1.20; CI 1.10, 1.32; p<0.001); and the adjusted odds were increased to 30% among children of mothers who were over 35 years. Another important variable is size of a child at birth. For instance, children who were very small at birth compared to very large at birth, had 39% lower odds of consuming ORS among diarrheal children (AOR = 0.61; CI 0.48, 0.78; p<0.001). Another important variable is place of first treatment sought for diarrheal children. That is children who first sought treatment in private hospital compared to government hospital had 52% lower odds of ORS consumption.

**Table 3:** Adjusted odds ratio for an association of covariates with ORS, Zinc and combined ORS + Zinc treatment among diarrhoeal children in India, 2019–21.

		ORS %		Total		Zinc %		Total		ORS + Zinc %		Total	
		ORS				Zinc				ORS + Zinc			
		Sig.	Exp(B)	95% C.I.for EXP(B)		Sig.	Exp(B)	95% C.I.for EXP(B)		Sig.	Exp(B)	95% C.I.for EXP(B)	
				Lower	Upper			Lower	Upper			Lower	Upper
Age of Mother	15–24												
	25–34	<0.001	1.20	1.10	1.32	0.016	1.12	1.02	1.23	<0.001	1.23	1.11	1.36
	35+	0.003	1.30	1.09	1.53	0.003	1.29	1.09	1.52	<0.001	1.40	1.17	1.67
Highest educational level	No education												
	Primary	0.180	0.92	0.81	1.04	0.558	1.04	0.91	1.18	0.906	0.99	0.86	1.14
	Secondary	0.179	1.07	0.97	1.19	0.310	1.06	0.95	1.18	0.756	1.02	0.91	1.14
	Higher	0.373	1.07	0.92	1.25	0.200	1.11	0.95	1.30	0.216	1.11	0.94	1.32
Type of place of residence	Urban												
	Rural	0.817	1.01	0.91	1.13	0.888	1.01	0.91	1.12	0.719	1.02	0.91	1.15
Wealth Index	Rich												
	Middle	0.424	0.95	0.85	1.07	0.981	1.00	0.89	1.13	0.770	0.98	0.86	1.12
	Poor	0.072	0.90	0.80	1.01	0.035	0.88	0.79	0.99	0.007	0.84	0.74	0.95
Currently breastfeeding	No												
	Yes	0.164	1.07	0.97	1.17	0.440	1.04	0.95	1.14	0.403	1.04	0.94	1.15
Sex of child	Male												
	Female	0.144	0.95	0.88	1.02	0.783	0.99	0.92	1.07	0.276	0.96	0.88	1.04
Birth weight	Very large												
	Large	<0.001	0.69	0.58	0.83	<0.001	0.56	0.47	0.66	<0.001	0.50	0.42	0.59
	Average	<0.001	0.61	0.53	0.71	<0.001	0.49	0.43	0.56	<0.001	0.45	0.39	0.51
	Low birth weight	<0.001	0.62	0.52	0.75	<0.001	0.50	0.42	0.60	<0.001	0.46	0.38	0.55
	Very low birth weight	<0.001	0.61	0.48	0.78	<0.001	0.47	0.37	0.60	<0.001	0.41	0.31	0.54
Age of child (in months)	> = 12												
	13–23	<0.001	1.92	1.74	2.13	0.003	1.17	1.05	1.30	<0.001	1.40	1.25	1.57
	24–35	<0.001	1.79	1.60	2.00	0.001	1.23	1.09	1.38	<0.001	1.40	1.24	1.60
	36–47	<0.001	1.99	1.75	2.26	<0.001	1.36	1.20	1.55	<0.001	1.62	1.41	1.86
	48–59	<0.001	1.77	1.54	2.03	0.001	1.27	1.11	1.46	<0.001	1.51	1.30	1.76
Caste	General												
	Scheduled caste/Scheduled tribe	0.676	0.98	0.87	1.10	<0.001	1.33	1.18	1.50	<0.001	1.36	1.19	1.55
	Other Backward Castes	0.001	0.83	0.74	0.92	0.185	1.08	0.96	1.21	0.379	1.06	0.93	1.20
Religion	Hindu												
	Muslim	0.282	1.07	0.95	1.21	0.111	1.10	0.98	1.25	0.066	1.13	0.99	1.29
	Others	0.008	0.80	0.68	0.94	0.587	0.96	0.81	1.12	0.288	0.91	0.76	1.08
Birth order	1												
	2	0.932	1.00	0.92	1.10	0.605	1.03	0.93	1.13	0.713	1.02	0.92	1.13
	3	0.562	1.04	0.92	1.17	0.417	0.95	0.84	1.08	0.638	0.97	0.85	1.11
	4+	0.997	1.00	0.87	1.15	0.129	0.89	0.77	1.03	0.062	0.86	0.74	1.01
Indian regions	South												
	Central	0.062	0.86	0.73	1.01	0.039	0.84	0.72	0.99	0.033	0.83	0.70	0.98
	East	0.942	1.01	0.85	1.18	0.001	0.76	0.65	0.90	0.007	0.79	0.66	0.94
	Northeast	0.004	1.37	1.10	1.69	0.002	0.72	0.58	0.88	0.046	0.80	0.64	1.00
	West	0.792	0.98	0.82	1.17	<0.001	0.71	0.60	0.85	0.001	0.72	0.60	0.87
	North	0.077	0.86	0.72	1.02	<0.001	0.65	0.55	0.77	<0.001	0.66	0.55	0.79
Place first sought treatment for diarrhoea	Government												
	Private	<0.001	0.48	0.44	0.53	<0.001	0.66	0.61	0.72	<0.001	0.60	0.55	0.66
	Others	<0.001	0.22	0.20	0.25	<0.001	0.30	0.27	0.33	<0.001	0.25	0.22	0.28
Diarrhoea in last 2 weeks: amount offered to drink	Nothing to drink												
	Less	0.021	1.27	1.04	1.55	0.001	1.45	1.16	1.83	<0.001	1.67	1.28	2.19
	Same as before/more	0.003	1.37	1.11	1.68	0.095	1.22	0.97	1.54	0.008	1.45	1.10	1.90
Diarrhoea in last 2 weeks: amount offered to eat	Less	<0.001	1.64	1.41	1.90	0.001	1.31	1.11	1.55	<0.001	1.44	1.19	1.75
	About the same/more	<0.001	1.40	1.20	1.64	<0.001	1.45	1.22	1.73	<0.001	1.58	1.30	1.94

Surprisingly, the logistic regression results for the other two dependent variables such as consumption of Zinc and ORS +Zinc combined by diarrheal children were similar. For instance, as age of mother and age of child increased, the odds of consumption of Zinc and ORS + Zinc combined also increased. Similarly, as in case of ORS consumption among children who first sought treatment in private hospitals compared to government hospitals, the lower odds of consumption observed both in case of Zinc and Zinc + ORS combined among diarrheal children. To be specific, lower odds of consumptions of Zinc and ORS + Zinc combined among diarrheal children were 34% and 40% respectively (AOR = 0.66; CI 0.61, 0.72; p<0.001; AOR = 0.60; CI 0.55, 0.66; p<0.001).

## Discussion

The latest National Family Health Survey data revealed that only 61% and 31% of the children were given ORS and Zinc respectively for treatment of diarrhoea. However, combined ORS + Zinc treatment was only around 24%. The treatment of ORS, Zinc, and combined ORS + Zinc supplementations were significantly higher among younger children, children of 24–35 age group mothers, children from rich wealth index groups, children who first sought treatment in government hospital and increased amount of food and drink offered during diarrhoea period. The adjusted odds ratios from logistic regression results also showed that consumption of ORS among diarrhoeal children under five years of age depends upon size of a child at birth. For instance, children who were very small size at birth (low birth weight) compared to very large at birth, had 39% lower odds of consuming ORS among diarrheal children. The inclusion of birth weight is important because low birth weight is related to preterm delivery or intra uterine growth restriction and this has been linked to an increased risk of insulin resistance or respiratory infections or both. Respiratory infections prevalence is found to be increasing the risk of diarrhoea [23]. The logistic regression adjusted AORs are similar with Zinc and ORS + Zinc supplementation for all most all variables.

We found that first seeking advice or treatment from private health facilities for the current episode of diarrhoea was 50%, and similarly, use of ORS, Zinc, and combined ORS + Zinc supplementations were much lower in private facilities compared with government health facilities. For instance, children who first sought treatment in private facilities compared to government facilities had 40% lower odds of ORS+Zinc consumption (Table 3). These results contradict the common belief that in India there is a widespread assumption that the quality of care provided in the private sector is better, competent and accountable as that compared to the public sector and hence, more women are expected to take their children for diarrheal treatment to private facilities. The review of the literature on the performance of the public and private sector, in low and middle-income countries including India, found that the private sector being more accountable, efficient or medically effective as compared to the public sector. The public sector was found to be lacking timeliness and hospitality towards patients [24].

The quality of care provided through public and private providers in low and middle-income country such as India has been a longstanding and highly debatable topic of discussion in the global health arena. The advocates of private sector consider it as the major healthcare provider and this sector to be more competent and more receptive to patients demand due to increased market competition [25]. In contrast, the public sector advocates bring out the inequalities in access to health care in the private sector due to the high cost of healthcare. To have a better understating of whether family income and mother’s education make any difference in the choice of place for children’s diarrhoea treatment, a separate logistic regression analysis was conducted using place of ORS+Zinc treatment (Public-Private) for children’s diarrhoea as a dependent variable, to assess the impact of education of mothers and household wealth after controlling for important confounding factors. Surprisingly, the household wealth index was not at all significant in differentiating the choice of treatment in public versus private facilities after controlling for confounding factors. Similarly, education of the mothers was also not a significant factor except in those mothers with university education, compared to mothers with no education (Table 1 in S1 Appendix). A paper published last year especially to assess the difference in maternal and neonatal quality of care in India, the authors found that public facilities did better than private facilities in the outcome indicators [26]. Similarly, other researchers in India have also found that the quality of public sector healthcare facilities have shown improvement in terms of physical infrastructure, the availability of essential equipment, enhanced accessibility, increased staff appointments, notably auxiliary nurse midwives (ANM), and overall service delivery strengthening, facilitated by increased funding under the National Health Mission (NHM) over the past two decades [27, 28]. Hence, education, income and whether women lived in rural or urban areas did not make any difference in seeking the diarrheal treatment for their children (Table 3).

To throw more light on public health system in India especially in the context of ORS and Zinc treatment for diarrheal children, the government programs are briefly highlighted. For instance, Ministry of Health and Family Welfare, Government of India, first launched the National Diarrheal Diseases Control Programme in 1978. The purpose of the programme was to prevent deaths due to dehydration caused by diarrheal diseases among children less than 5 years of age. The program was successful in reducing child mortality from diarrhoea by 50% between 1981 and 1990. However, the government shifted its focus in 1985–1986 to strengthen case management of diarrhoea for children of less than 5 years of age and consequently the Oral Rehydration Therapy (ORT) programme was introduced. Later, ORT programme became part of child survival and safe motherhood program in 1992 and reproductive and child health (RCH) programme in 1997. Another significant move was the launch of National Rural Health Mission (NRHM) in April 2005, a flagship program by the government of India, to tackle the high burden of maternal, neonatal and child morbidity and mortality among India’s rural populations. More recently, the Government of India (GOI) named the program National Health Mission (NHM), to also include coverage of urban poor. NHM reaches out to underserved areas through health programs such as the Village Health and Nutrition Days. During these nutrition and health education sessions organized by the auxiliary nurse midwife and Anganwadi workers, the use of ORT is popularized among the community members.

Despite massive efforts by Government of India in controlling children’s deaths due to diarrhea, the efforts have not achieved desired results. Hence, the Government of India introduced a new Intensified Diarrhea Control Fortnight (IDCF) program in 2014 [29]. The government of India realized that meeting the childhood mortality target of 23 per 1,000 live births by 2025 as the prime goal of National Health Policy of 2019 will not be possible. Hence, national efforts to control childhood diarrhea must be intensified to achieve the coverage of both ORS and Zinc to reach the target of 90 percent by 2025 as per the India Action Plan for Pneumonia and Diarrhea (IAPPD). However, from 2015–16 to 2019–21, at the national level, ORS coverage increased from 50.6 percent (NFHS 4) [30] to 60.6 percent (NFHS 5) and Zinc coverage from 20.3 percent (2005–06) (NFHS 3) [31] to 30.5 percent during 2015–16 (NFHS 4). To increase the coverage of ORS and Zinc, the Ministry of Health and Family Welfare in close collaboration with State / Union Territory (UT) governments has been implementing Intensified Diarrhea Control Fortnight (IDCF). For this purpose, the Ministry of Health and Family Welfare, Government of India, introduced Operational Guidelines to achieve “zero” childhood deaths due to diarrhea across all States/UTs [32]. The specific set of activities for prevention and control of deaths due to dehydration from diarrhea include- intensification of advocacy and awareness generation for diarrhea management, strengthening service provision for diarrhea case management, establishment of ORS-Zinc corners, prepositioning of ORS and Zinc by Accredited Social Health Activists (ASHAs) in households with under-five children and awareness generation activities for hygiene and sanitation. More importantly, the government organizes an Intensified Diarrhea Control Fortnight for two weeks from 13th– 27th June 2022, to make sure that every family gets an ORS kit. Special focus was on preparatory activity to address potentially high incidence of diarrhea during the summer/monsoon season and floods/natural calamity to be accorded to the high priority areas and vulnerable communities. The IDCF strategy is three folds: 1) Improved availability and use of ORS at households, 2) Facility level strengthening to manage cases of dehydration and 3) Enhanced advocacy and communication on prevention and control of diarrhea through IEC campaign. The government decision to involve of ASHAs and Auxiliary Nurse Midwives (ANMs) to undertake village child health activities for the early detection and prompt treatment of diarrhea cases in the community is important because community level activities provide the last mile connectivity [32]. The Guidelines also include continuous efforts of orientation of all stakeholders at all levels through meetings or video conference by State NHM to sensitize for planning of IDCF. It also provides agenda items to be covered during meetings. The orientation is expected to focus on explanation on distribution of ORS, management of diarrhea, supportive supervision, IEC material and plan of activities to all frontline workers. The Guidelines include module for technical orientation on childhood diarrhea control toolkit.

The Operational Guidelines noted that the weak supply chain for procurement of essential drugs such as ORS and Zinc potentially exposed patients to financial hardships and diminished public trust in the health system. A serious policy response was recommended to address gaps in drug and equipment procurement in all states and UTs. This necessitated a thorough review of the then existing public health drug supply chain system model, studying best practices in other states and countries redesigning for ensuring availability of essential drugs required for the patients at all public health facilities. The Operational Guidelines also focus on the framework for accountability and in enhancing the program’s capacity to monitor and evaluate the results. The Guidelines show how to improve measurement of the core indicators and how to be strengthening children’s diarrhoea information systems. Evaluation of current initiatives to explore the potential of information and communication technologies to improve the speed and accuracy of reporting, particularly at community level, and scaling up where there is evidence of their effectiveness; and support to build country capacity to monitor, review and act on data. The Guidelines also discuss various tools and covers the use of data for monitoring purposes and strengthening reporting mechanisms at all levels of the health system.

It was also found that only one-third of the children were offered to drink or eat about the same amount during diarrhoea (Table 3). It is necessary to practice continued feeding for diarrhoeal children. Diarrhoeal children usually tend to suffer from decreased intake of essential nutrients, and this may have serious consequences. This is especially the case among malnourished children from developing or less developed countries. These children tend to suffer from double jeopardy of malnutrition and diarrhoea and hence, at least they should be provided with adequate food to eat and drink during diarrhoea. This is especially important among children suffering from acute diarrhoea.

The results indicate that that younger-age children are more likely to develop diarrheal diseases than older-age children. These results corroborate with the findings of other studies in Africa and as argued by other researchers that lower prevalence of diarrheal illness in the older children could be due to acquired natural immunity [33, 34]. However, the present study found that younger age group children received less percentage of ORS, Zinc also combined ORS + Zinc supplementations compared to older children. Under these circumstances if diarrhoeal children are not treated for dehydration and electrolyte depletion, then the serious consequence of death is inevitable among these children. World Health Organization and the United Nations International Children’s Emergency Fund have initiated the Comprehensive Global Action Plan on Pneumonia and Diarrhoea (GAPPD) in 2013 [35]. One of the goals of the plan is to reduce the incidence of diarrhoeal disease by 75% in children under five years of age by 2025. However, this study revealed a low prevalence of ORS, Zinc and combined ORZ + Zinc supplementations, especially among younger children [34, 35] is to be noted and Indian policy makers and programmers should address this issue as soon as possible to meet the Global Action plan goals by 2025.

There are some limitations of the data. For instance, the frontline workers distribute ORS and Zinc packets for every family, but it is not clear whether the care givers have the correct knowledge on the preparation of solution. Similarly, the data has been collected on birthweight for all live births. Babies born alive with a birthweight of less than 2500 grams are considered Low birth weight infants. However, the anecdotal evidence suggest that the measurement of birthweight is not done using digital weighing machine. Hence, the quality of data on correct measurement can be questionable. The study’s findings need to be interpreted with caution. The data is from a cross-sectional survey, which limits the scope of establishing causality among variables. Moreover, the predictors included in the analysis based on the availability of information collected in the survey and may not be comprehensive in understanding ORS and Zinc treatment for diarrhoeal children at the facilities since this is not the sole purpose of the survey. For instance, there is no information on knowledge of ORS and Zinc treatment for diarrhoea among the mothers.

## Conclusions

To conclude, the Indian children of younger age group mothers and younger age group children are significantly consuming less ORS, Zinc and combined ORS + Zinc supplementation for treatment of diarrhoea. Those children who sought first treatment at private facilities had low use of ORS, Zinc and combined ORS + Zinc treatment. The flagship program of the government, IDCF, should not only involve ASHAs for distribution of ORS and Zinc packets for every family and should demonstrate the preparation of solution and making sure that the care givers are comfortable with the execution. Similarly, ANMs should conduct IDCF meeting in their Sub Centres and Village Health and Nutrition Meetings to discuss on prevention and control of diarrhoea, especially involving care givers of under-five children. ANMs should also emphasize continued feeding during diarrhoea period, hand washing and use of toilets for defecation. In addition, the private health care providers especially in rural areas should be sensitized about IDCF program and the government of India should consider periodic training programs to the private health care providers on ORS and Zinc treatment to prevent and control of diarrhoea among children.

The current approaches to programming may have to be re-examined. For instance, applying advanced methodologies to more robust hotspot identification and interventions targeted to identified populations in geographic areas may greatly improve the coverage of the ORS and Zinc in India. It would be also useful to sensitize ASHAs and other stake holders to address any barriers against disadvantaged communities’ in accessing and of using ORS and Zinc treatment for children’s diarrhoea. Overall, direct information to women and couples using digital tools and technology needs to be explored to improve access accurate information and for follow-up.

## Supporting information

S1 Appendix Adjusted odds ratio for an association of covariates with type of treatment facility (private/public) among diarrhoeal children in India, 2019–21. (DOCX)

S1 Checklist STROBE statement—checklist of items that should be included in reports of *cross-sectional studies*. (DOC)
