# Supplementary material for: Oral rehydration therapy and Zinc treatment among diarrhoeal children in India: Exploration from latest cross-sectional National Family Health Survey
Source: PLoS One. 2024 Oct 3;19(10):e0307657. doi: 10.1371/journal.pone.0307657 (PMC11449306; doi:10.1371/journal.pone.0307657)
Supplement: S1 Appendix — (DOCX) [file pone.0307657.s001.docx]

Appendix 1: Adjusted Odds Ratio for an association of covariates with type of

treatment facility (private/public) among diarrhoeal children in India, 2019-21

| Treatment in Private Facilities | | | | | |
| --- | --- | --- | --- | --- | --- |
|  |  | Sig. | Exp(B) | 95% C.I.for EXP(B) |  |
|  |  |  |  | Lower | Upper |
| Age of Mother | 15-24 |  |  |  |  |
|  | 25-34 | 0.155 | 0.875 | 0.728 | 1.052 |
|  | 35+ | 0.081 | 0.740 | 0.528 | 1.038 |
| Highest educational level | No education |  |  |  |  |
|  | Primary | 0.194 | 0.833 | 0.632 | 1.098 |
|  | Secondary | 0.961 | 0.995 | 0.798 | 1.239 |
|  | Higher | 0.021 | 1.451 | 1.059 | 1.990 |
| Wealth Index | Rich |  |  |  |  |
|  | Middle | 0.124 | 0.836 | 0.665 | 1.051 |
|  | Poor | 0.425 | 0.916 | 0.737 | 1.137 |
| Type of place of residence | Urban |  |  |  |  |
|  | Rural | 0.043 | 0.811 | 0.663 | 0.993 |
| Currently breastfeeding | No |  |  |  |  |
|  | Yes | 0.563 | 0.948 | 0.792 | 1.135 |
| Sex of child | Male |  |  |  |  |
|  | Female | 0.024 | 0.842 | 0.725 | 0.978 |
| Birth weight | Very large |  |  |  |  |
|  | Large | 0.169 | 0.813 | 0.604 | 1.092 |
|  | Average | 0.287 | 0.880 | 0.696 | 1.113 |
|  | Low birth weight | 0.503 | 1.120 | 0.804 | 1.561 |
|  | Very low birth weight | 0.322 | 0.799 | 0.513 | 1.246 |
| Age of child (in months) | >=12 |  |  |  |  |
|  | 13-23 | 0.001 | 1.446 | 1.169 | 1.789 |
|  | 24-35 | 0.949 | 1.008 | 0.800 | 1.269 |
|  | 36-47 | 0.138 | 0.826 | 0.642 | 1.063 |
|  | 48-59 | 0.001 | 0.634 | 0.485 | 0.829 |
| Caste | General |  |  |  |  |
|  | Scheduled caste/Scheduled tribe | 0.450 | 0.916 | 0.730 | 1.150 |
|  | Other Backward Castes | 0.015 | 1.301 | 1.052 | 1.610 |
| Religion | Hindu |  |  |  |  |
|  | Muslim | 0.708 | 1.045 | 0.830 | 1.316 |
|  | Others | 0.002 | 0.503 | 0.328 | 0.772 |
| Birth order | 1 |  |  |  |  |
|  | 2 | 0.574 | 0.948 | 0.788 | 1.141 |
|  | 3 | 0.124 | 1.218 | 0.947 | 1.566 |
|  | 4+ | 0.233 | 1.200 | 0.889 | 1.618 |
