# Supplementary material for: Oral rehydration therapy and Zinc treatment among diarrhoeal children in India: Exploration from latest cross-sectional National Family Health Survey
Source: PLoS One. 2024 Oct 3;19(10):e0307657. doi: 10.1371/journal.pone.0307657 (PMC11449306; doi:10.1371/journal.pone.0307657)
Supplement: S1 Checklist — (DOC) [file pone.0307657.s002.doc]

STROBE Statement—Checklist of items that should be included in reports of ***cross-sectional studies***

|  | Item No | Recommendation |
| --- | --- | --- |
| **Title and abstract** | 1 | (*a*) Indicate the study’s design with a commonly used term in the title or the abstract  **The study’s design is indicated both in the title and abstract.** |
|  |  | (*b*) Provide in the abstract an informative and balanced summary of what was done and what was found  **This is done.** |
| Introduction | | |
| Background/rationale | 2 | Explain the scientific background and rationale for the investigation being reported  **This is done.** |
| Objectives | 3 | State specific objectives, including any prespecified hypotheses  **Towards end of the background section, the purpose of the study is mentioned**. |
| Methods | | |
| Study design | 4 | Present key elements of study design early in the paper. **Done (please refer methods section)** |
| Setting | 5 | Describe the setting, locations, and relevant dates, including periods of recruitment, exposure, follow-up, and data collection. **Done (please refer first paragraph of methods section)** |
| Participants | 6 | (*a*) Give the eligibility criteria, and the sources and methods of selection of participants. **Done (please refer an *analytical sample* sub-section in methods Section)** |
| Variables | 7 | Clearly define all outcomes, exposures, predictors, potential confounders, and effect modifiers. Give diagnostic criteria, if applicable. **Done (please refer sub-section** outcome variable and predictor variables **in methods section)** |
| Data sources/ measurement | 8* | For each variable of interest, give sources of data and details of methods of assessment (measurement). Describe comparability of assessment methods if there is more than one group. **It is a secondary data source and measurement of variables is indicated in sub-sections *outcome variable and predictor variables* in methods section.** |
| Bias | 9 | Describe any efforts to address potential sources of bias. **It is a secondary data source, and limitations are indicated towards end of discussion section.** |
| Study size | 10 | Explain how the study size was arrived at. **Done (please refer an *analytical sample* sub-section in methods Section)** |
| Quantitative variables | 11 | Explain how quantitative variables were handled in the analyses. If applicable, describe which groupings were chosen and why. **Done (please refer an *analytical sample* sub-section in methods Section)** |
| Statistical methods | 12 | (*a*) Describe all statistical methods, including those used to control for confounding. **Done (please refer a *data analysis* sub-section in methods Section)** |
|  |  | (*b*) Describe any methods used to examine subgroups and interactions. **N/A** |
|  |  | (*c*) Explain how missing data were addressed. **Pairwise method is used.** |
|  |  | (*d*) If applicable, describe analytical methods taking account of sampling strategy. **N/A** |
|  |  | (*e*) Describe any sensitivity analyses. **N/A** |
| Results | | |
| Participants | 13* | (a) Report numbers of individuals at each stage of study—eg numbers potentially eligible, examined for eligibility, confirmed eligible, included in the study, completing follow-up, and analysed. **Number of cases are provided in the tables.** |
|  |  | (b) Give reasons for non-participation at each stage. **Inclusion/Exclusion criteria** |
|  |  | (c) Consider use of a flow diagram. **This was not necessary since we have mentioned clearly inclusion and exclusion criteria.** |
| Descriptive data | 14* | (a) Give characteristics of study participants (eg demographic, clinical, social) and information on exposures and potential confounders. **Done (please refer a background sub-section in results section)** |
|  |  | (b) Indicate number of participants with missing data for each variable of interest. **After inclusion and exclusion criteria no missing cases were observed.** |
| Outcome data | 15* | Report numbers of outcome events or summary measures. **Done (please refer table 2)** |
| Main results | 16 | (*a*) Give unadjusted estimates and, if applicable, confounder-adjusted estimates and their precision (eg, 95% confidence interval). Make clear which confounders were adjusted for and why they were included. **Used multivariate analysis for controlling for confounders (please see table 2 for unadjusted and table 3 for adjusted estimates).** |
|  |  | (*b*) Report category boundaries when continuous variables were categorized. **Done (please see tables)** |
|  |  | (*c*) If relevant, consider translating estimates of relative risk into absolute risk for a meaningful time period. **N/A** |
| Other analyses | 17 | Report other analyses done—eg analyses of subgroups and interactions, and sensitivity analyses. (**please see Appendix table 1**) |
| Discussion | | |
| Key results | 18 | Summarise key results with reference to study objectives. **Done** |
| Limitations | 19 | Discuss limitations of the study, taking into account sources of potential bias or imprecision. Discuss both direction and magnitude of any potential bias. **Done (please refer last paragraph of discussion section)** |
| Interpretation | 20 | Give a cautious overall interpretation of results considering objectives, limitations, multiplicity of analyses, results from similar studies, and other relevant evidence. **Done (please refer discussion section)** |
| Generalisability | 21 | Discuss the generalisability (external validity) of the study results. **The results are based on a national sample.** |
| Other information | | |
| Funding | 22 | Give the source of funding and the role of the funders for the present study and, if applicable, for the original study on which the present article is based. **We are using secondary data source, and no funding is received.** |

*Give information separately for exposed and unexposed groups.

**Note:** An Explanation and Elaboration article discusses each checklist item and gives methodological background and published examples of transparent reporting. The STROBE checklist is best used in conjunction with this article (freely available on the Web sites of PLoS Medicine at http://www.plosmedicine.org/, Annals of Internal Medicine at http://www.annals.org/, and Epidemiology at http://www.epidem.com/). Information on the STROBE Initiative is available at www.strobe-statement.org.
